# An Overview of Cushing’s Syndrome Caused by Ectopic Adrenocorticotropin Secretion

**DOI:** 10.3390/jcm15208192

**Published:** 2026-10-21

**Authors:** Ioannis Ilias, Viviana Maggio, Sorina Ispas, Karolina Hoffmann, Anna Paczkowska, Ashot Avagimyan, Imran Rashid Rangraze, Adil Farooq Wali, Sirajunisa Talath, Syed Arman Rabbani, Bhoomendra A. Bhongade, Shakta Mani Satyam, Manfredi Rizzo, Mohamed El-Tanani

**Affiliations:** 1 Department of Endocrinology, Hippokration Hospital, 115 27 Athens, Greece; iliasendo@hippocratio.gr; 2 School of Medicine, PROMISE Department of Health Promotion Sciences Maternal and Infantile Care, Internal Medicine and Medicinal Specialties, University of Palermo, 90133 Palermo, Italy; 3 Department of Anatomy, Faculty of General Medicine, Ovidius University, 900470 Constanta, Romania; sorina.ispas@365.univ-ovidius.ro; 4 Department and Clinic of Internal Diseases and Metabolic Disorders, Poznan University of Medical Sciences, 61-701 Poznań, Poland; 5 Department of Pharmacoeconomics and Social Pharmacy, Poznan University of Medical Sciences, 61-701 Poznań, Poland; 6 Department of Anatomical Pathology & Clinical Morphology, Mkhitar Heratsi Yerevan State Medical University, Yerevan 0025, Armenia; 7 Isfahan Cardiovascular Research Centre, Cardiovascular Research Institute, Isfahan University of Medical Sciences, Isfahan 81465-1148, Iran; 8 RAK College of Medical Sciences, RAK Medical and Health Sciences University, Ras Al Khaimah 11172, United Arab Emirates; 9 RAK College of Pharmacy, RAK Medical and Health Sciences University, Ras Al Khaimah 11172, United Arab Emiratessatyam@rakmhsu.ac.ae (S.M.S.)

**Keywords:** ectopic Cushing’s syndrome, ectopic ACTH, neuroendocrine neoplasms, hypercortisolism, paraneoplastic syndrome

## Abstract

Ectopic Cushing’s syndrome (ECS) causes 10% to 20% of all cases of endogenous hypercortisolism. It is caused by the aberrant secretion of adrenocorticotropic hormone (ACTH) or corticotropin-releasing hormone (CRH) due to the presence of an ectopic tumor. The latter is usually a neuroendocrine (NEN) tumor (mostly thoracic NENs such as bronchial NEN neoplasms, thymic NEN neoplasms, small cell lung carcinoma (SCLC), or pancreatic NEN neoplasms). Clinical presentation is heterogeneous: an aggressive phenotype, typically driven by rapidly growing, poorly differentiated tumors such as small-cell lung carcinoma, manifests with severe symptoms of acute onset, predominant myopathy, resistant arterial hypertension, hypokalemia, and severe hyperpigmentation, usually without the centripetal obesity typical of Cushing’s syndrome; conversely, an indolent phenotype, more often related to well-differentiated neuroendocrine neoplasms such as bronchial carcinoids, may present with the classic centripetal fat distribution, facial plethora, and purple striae also seen in Cushing’s disease, making clinical distinction between the two difficult. The diagnosis of ECS can be cumbersome and time-consuming. Once hypercortisolism is confirmed, the most critical step is to distinguish patients with an ectopic source of ACTH from those with pituitary adenoma. Dynamic endocrine tests such as the high-dose dexamethasone suppression test, CRH stimulation tests, desmopressin tests, and bilateral inferior petrosal sinus sampling (BIPSS) can be helpful in the differential diagnosis. BIPSS is considered the gold standard for distinguishing a pituitary from a non-pituitary source of ACTH: the absence of a central-to-peripheral ACTH gradient supports, but does not by itself localize or confirm, an ectopic source. Tumor localization is achieved by a multimodality imaging protocol, including initial high-resolution computed tomography (CT) scans, followed by functional imaging, mainly Gallium-68-labeled somatostatin receptor positron emission tomography (PET)/CT scans. However, in 10–20% of these patients, the tumors cannot be localized. The treatment of ACTH-dependent hypercortisolism is two-fold: one is to immediately control hypercortisolism to prevent life-threatening complications (opportunistic infections and thromboembolism), and the second is to definitively treat the tumor. Medical treatment with steroidogenesis inhibitors, including metyrapone, ketoconazole, and osilodrostat, or surgical management (bilateral adrenalectomy) can be effective in immediately controlling hypercortisolism. Definitive surgical resection of the tumor is the hoped-for final treatment. The prognosis is variable, depending on the nature of the underlying neoplasm, ranging from excellent in patients with resected bronchial carcinoids to dismal in those with SCLC.

## 1. Introduction

Cushing’s syndrome (CS) is described as a constellation of clinical manifestations resulting from hypercortisolism. The most common cause of CS in clinical practice is steroid use in the management of inflammatory/autoimmune diseases. However, endogenous CS is a serious illness with an incidence rate of two to eight cases of CS per million individuals annually [1,2]. The long-term effects of hypercortisolism involve all body systems, resulting in significant morbidity, including hyperglycemia, protein loss, hypertension, immune suppression, emotional, and cognitive changes, as well as mortality [3,4]. Endogenous CS is classified broadly into two groups based on its underlying pathophysiology: ACTH-independent and ACTH-dependent states [5]. ACTH-independent CS, occurring in about 20–30% of cases, is due to spontaneous cortisol secretion by an adrenal adenoma, carcinoma, or bilateral hyperplasia and causes suppression of pituitary ACTH secretion [1]. Most cases (60–80%) of ACTH-dependent CS are due to excess ACTH secretion by a benign corticotroph (ACTH-secreting) adenoma of the pituitary gland (causing Cushing’s disease; CD) [1]. However, approximately 10–20% of patients with ACTH-dependent CS are classified as having ectopic Cushing’s syndrome (ECS) [6,7]. In this rare paraneoplastic syndrome, ACTH is secreted by a non-pituitary tumor [6]. In extremely rare instances (<1%), ECS results from ectopic secretion of corticotropin-releasing hormone (CRH), which then causes pituitary corticotrophs to secrete inordinate amounts of ACTH [1,8]. The diagnostic and therapeutic journey for a patient with an ECS is often a hazardous one, replete with diagnostic uncertainty, the frustrating pursuit of an elusive tumor, and the need to treat threatening complications of life-threatening severe hypercortisolism [9]. Throughout this review, ECS is used as the overarching clinical term for paraneoplastic, ACTH-dependent Cushing’s syndrome due to a non-pituitary tumor, while EAS (and the equivalent expressions ectopic ACTH syndrome/ectopic ACTH secretion) refer specifically to the much more common subset in which the tumor itself secretes ACTH, as distinct from the rare ectopic CRH syndrome described in Section 2.1. In keeping with current World Health Organization nomenclature, the terms neuroendocrine neoplasm (NEN) and neuroendocrine tumor (NET) are used interchangeably, and carcinoid is retained only where it refers to well-differentiated bronchial or thymic NENs, reflecting continued clinical usage. In this narrative review, we will present the complex territory of ECS, from its most fundamental pathophysiology to recent aspects of diagnosis and management, with the aim of providing a comprehensive roadmap for the clinician faced with this formidable challenge.

## 2. Pathophysiology and Etiology

### 2.1. Disruption of the Hypothalamic–Pituitary–Adrenal Axis

The pathophysiology of ECS relies on dysregulation of the fine-tuned HPA axis. Under normal conditions, the hypothalamus releases CRH that stimulates pituitary corticotroph cells to release and secrete ACTH [10]. ACTH then acts upon the adrenal cortex to stimulate the synthesis and secretion of cortisol. Cortisol closes the feedback loop by inhibiting ACTH and CRH secretion at both the pituitary and hypothalamic levels (negative feedback), thereby maintaining homeostasis [10,11].

In ECS, an autonomously ACTH-producing and -secreting non-pituitary tumor leads to the adrenal glands secreting abnormal amounts of cortisol [6]. This cortisol flood strongly suppresses the physiological production of hypothalamic CRH and pituitary ACTH under normal circumstances [10]. The tumor-derived ACTH is resistant to this feedback suppression, leading to persistent and uncontrolled hypercortisolism [12].

ACTH released by the ectopic tumors is derived from the same precursor molecule as pituitary ACTH, pro-opiomelanocortin (POMC) [10,13]. However, post-translational processing of POMC in these tumors can be abnormal and lead to the release of not only mature ACTH (1–39) but also larger, partially processed precursors like “big ACTH” and other POMC-related peptides [14,15]. This altered processing is of diagnostic significance because some immunoassays will not be able to detect such precursor molecules efficiently and therefore may lead to an underestimation of the actual levels of corticotropic peptides [14]. Molecular studies provide evidence that ectopic aberrant activation of the POMC gene promoter, possibly through hypomethylation or the action of ectopic transcription factors that are not typically expressed in these tissues, is responsible for this pathological expression [12,16].

In rare instances of ectopic CRH syndrome, a tumor secretes CRH, causing secondary activation of pituitary corticotrophs, which are hyperplastic and inappropriately secrete excess ACTH [8,17]. This condition is challenging to diagnose as it biochemically mimics pituitary CD, being in some instances sensitive to CRH stimulation tests and even showing a central-to-peripheral gradient on bilateral inferior petrosal sinus sampling (BIPSS) [5].

### 2.2. Etiology: Causal Tumor Spectrum

Ectopic secretion of ACTH is almost exclusively a feature of neuroendocrine neoplasms (NENs), which can arise from a multitude of anatomical sites [6]. While many types of tumors have been implicated, some select forms account for the vast majority of cases (Table 1).

Frequencies are derived from multiple large case series and may vary based on referral patterns (e.g., endocrinology vs. oncology centers). These wide ranges also reflect differences in case ascertainment (systematic biochemical screening of neuroendocrine neoplasm cohorts versus clinically driven endocrine referral series) and in whether occult, source-undiscovered cases are classified separately, which explains why single-center surgical series and multicenter endocrine registries often report divergent frequencies for the same tumor type.

Thoracic NENs are the most common source of ECS and account for over half of all cases [6,27]. Bronchial NENs, alternatively termed bronchial carcinoids, are the most typical cause in the majority of reported endocrine series of patients and may have an indolent clinical behavior [6,26,28]. In contrast, SCLC is associated with a very aggressive and rapidly progressing form of ECS [29]. Thymic NENs are also a notable but less common thoracic source, are aggressive in nature, and are associated with Multiple Endocrine Neoplasia Type 1 (MEN-1) syndrome [22].

Gastroenteropancreatic NENs (GEP-NETs), particularly those of pancreatic in origin, are the second most common category of tumors [23,30]. Pheochromocytomas, paragangliomas [25], and medullary thyroid carcinoma (MTC), typically in the context of MEN-2 [19], less often lead to ECS. A varied and constantly expanding spectrum of other rare tumors, including prostate, gastrointestinal tract, and ovarian tumors, has also been reported to lead to ECS [6,31].

One of the significant diagnostic challenges is the so-called occult tumor, for which, despite extensive and repeated investigation, the etiology of ectopic ACTH cannot be established in about 10–20% of cases [6,32]. Of these, most are ultimately found to be small, slow-growing bronchial NENs that may not become apparent until many years after the initial diagnosis [9].

## 3. Epidemiology

Endogenous CS is a rare disease, with its yearly incidence estimated to be between two and eight cases per million people [1]. ECS is responsible for about 10–20% of all such diagnoses and is therefore an extremely uncommon diagnosis [6]. Its frequency among large cohorts of patients with proven thoracic or gastroenteropancreatic NENs has been estimated at around 3.2% [30].

The distribution of ECS by age and sex is significantly different from that of Cushing’s disease. CD has a female predominance (up to 9:1), while ECS affects women and men almost equally [1]. Patients with ECS are also diagnosed at an older age compared to those with CD [33]. ECS from thymic NENs is an exception, as it presents at a younger mean age of approximately 35 years with male predominance [22].

One of the most significant features of ECS is the shorter duration of symptoms prior to diagnosis. A meta-analysis established the mean time to diagnosis for ECS as 14 months and for CD as 38 months [34]. This would most likely be due to the fact that most ECS cases present with more extreme and acutely manifesting symptoms, thereby prompting medical consultation earlier on [35].

## 4. Clinical Presentation

The clinical presentation of ECS is extremely variable, from an indolent course akin to CD to a fulminant, life-threatening condition [6,35]. The variability is largely dependent on the character of the underlying tumor and the degree of hypercortisolism [9]. Presentation can be broadly categorized into two phenotypes: the indolent and the aggressive subtypes [35].

The indolent subtype is typically associated with highly differentiated NENs, such as bronchial NENs (carcinoids) [6]. Patients with this subtype typically have an insidious onset of the classic symptoms and signs of CS, such as centripetal obesity, facial plethora, purple striae, and glucose intolerance, so that clinical differentiation from Cushing’s disease becomes clinically impossible at presentation [36].

Conversely, the aggressive phenotype is characteristic of poorly differentiated malignancies like SCLC or some high-grade NENs [35]. Presentation is generally acute in these patients, and the clinical presentation is dominated by the intense catabolic effect of profound hypercortisolism [6]. Patients may also have severe proximal myopathy and weight loss, unlike the usual weight gain associated with CD [37]. Other characteristic features of severe, rapid-onset ECS are shown in Table 2.

## 5. Diagnosis and Differential Diagnosis

**Figure 1:**
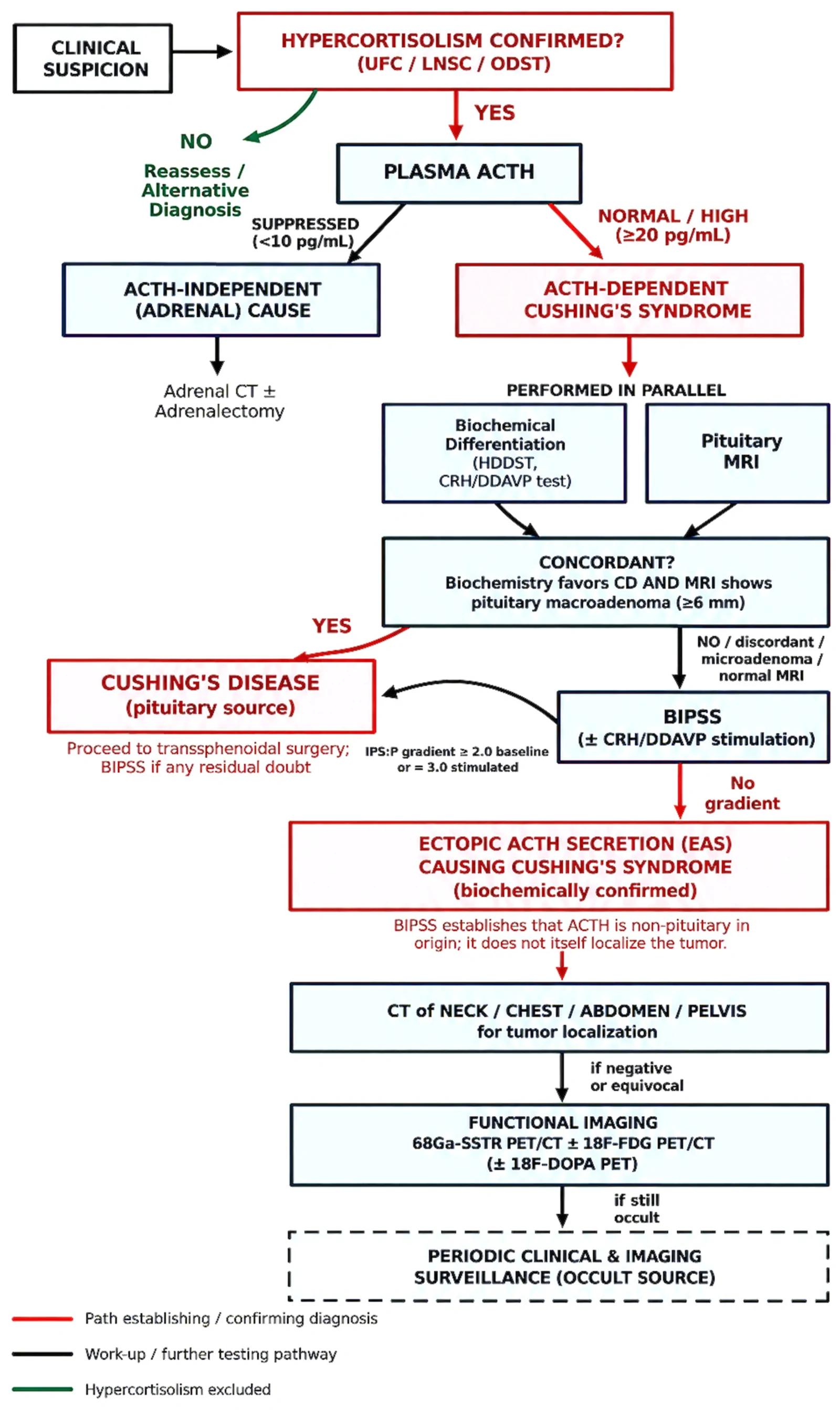
Diagnostic steps for the evaluation of possible Cushing’s syndrome and localization, if caused by ectopic ACTH secretion. The figure was revised to accurately reflect the integration of biochemical testing, pituitary imaging, and BIPSS, per reviewer comments; a positive pituitary MRI no longer establishes Cushing’s disease without biochemical/BIPSS confirmation, and BIPSS is now shown as diagnostic of the source rather than of localization.

The diagnostic process in a patient with suspected ECS is a multifaceted, step-by-step process and potentially one of the more challenging problems for the practicing clinician [44]. It involves confirming hypercortisolism, establishing ACTH-dependence, differentiating the ectopic source from a pituitary one, and finally, localizing the culprit tumor [45] (Figure 1).

### 5.1. Confirmation of Hypercortisolism and Establishment of ACTH-Dependence

This initial diagnostic step comprises two sequential components: first, biochemical confirmation of endogenous hypercortisolism itself, and second, once hypercortisolism is confirmed, determination of whether ACTH secretion is driving it (i.e., establishing ACTH-dependence).

The initial step is to directly verify the presence of endogenous hypercortisolism [46]. This is usually performed using at least two initial screening tests, such as a 24 h urinary free cortisol (UFC) test, a late-night salivary cortisol (LNSC) test, or a 1 mg overnight dexamethasone suppression test (ODST) [1]. These studies are typically significantly abnormal in ECS, particularly in its severe forms, and the diagnosis of hypercortisolism is straightforward [47].

Following the development of hypercortisolism, the second step is the measurement of morning plasma ACTH to determine ACTH dependence [14]. In ECS, ACTH can be normal or, more often, extremely elevated (usually >20 pg/mL), whereas in ACTH-independent adrenal CS, it is suppressed (<10 pg/mL) [1]. Extremely high ACTH levels (>200–250 pg/mL) are more common in ECS than in CD, but with substantial overlap [37].

### 5.2. Differentiation of Ectopic from Pituitary Sources

This is the most crucial and often most difficult step in the diagnostic algorithm [44]. The distinction relies on exploiting the subtle differences in the pathophysiology of corticotroph adenomas versus ectopic ACTH-secreting tumors [12]. Pituitary adenomas in CD usually retain some degree of responsiveness to negative feedback and to physiological secretagogues, whereas ectopic tumors are generally more autonomous [48].

### 5.3. Non-Invasive Biochemical Tests for ECS

The non-invasive biochemical tests used in the evaluation of ECS are shown in Table 3.

Due to the imperfect accuracy of individual tests, a combined strategy is often employed [55]. A concordant result from multiple tests (e.g., lack of suppression to HDDST and no response to CRH/DDAVP) increases the diagnostic certainty for ECS [18].

### 5.4. Invasive Testing: Bilateral Inferior Petrosal Sinus Sampling (BIPSS)

When non-invasive tests are inconclusive or discordant with imaging, BIPSS is the gold standard for definitively differentiating a pituitary from an ectopic source of ACTH [56,57]. This procedure involves catheterizing the veins that directly drain the pituitary gland (the inferior petrosal sinuses) and simultaneously sampling blood from both sinuses and a peripheral vein for ACTH measurement, both at baseline and after stimulation with CRH or desmopressin [58]. A central-to-peripheral (IPS:P) ACTH gradient ≥2.0 at baseline or ≥3.0 after stimulation is diagnostic of Cushing’s disease [56]. The absence of such a gradient strongly indicates an ectopic source [58]. Importantly, BIPSS distinguishes a pituitary from a non-pituitary (ectopic) source of ACTH; it does not itself localize or confirm the ectopic tumor, which still requires dedicated anatomical and functional imaging (Section 5.5). In experienced hands, BIPSS has a sensitivity of over 94% and a specificity approaching 100% [56,59]. However, the procedure is technically demanding and carries risks [60]. Pitfalls of BIPSS include anomalous venous drainage, which can lead to false-negative results (suggesting ECS in a patient with CD), and cyclic CS, where sampling during a quiescent phase can be misleading [61,62]. Measuring prolactin concurrently can help confirm correct catheter placement and adequacy of pituitary venous sampling [57,63].

### 5.5. Tumor Localization

Once an ectopic source of ACTH is confirmed biochemically, the quest to find the tumor begins [44]. This often requires a multimodal imaging approach [64].

#### 5.5.1. Anatomical Imaging

The initial imaging modality of choice is a high-resolution, thin-slice, contrast-enhanced computed tomography (CT) of the neck, chest, and abdomen [65,66]. This is because the majority of ectopic sources are located in the thorax or abdomen [19]. CT identifies the tumor in approximately 66% of cases [64]. Magnetic resonance imaging (MRI) is a valuable complementary tool, particularly for visualizing the pancreas, liver, and mediastinum, but is generally less sensitive for small pulmonary lesions [65].

#### 5.5.2. Functional Imaging

When anatomical imaging is negative or equivocal, functional imaging (nuclear medicine) techniques are employed [64]. These modalities exploit the specific biological properties of NENs [67] (Table 4).

Despite this extensive imaging armamentarium, the primary tumor remains occult in 10–20% of patients with biochemically proven ECS [6,18]. These patients require long-term follow-up with periodic reimaging, as the tumor may declare itself over time [32].

## 6. Treatment

Treatment of ECS is multidisciplinary and ideally performed in a specialized center [43]. The therapeutic goals are threefold: (a) rapid and effective control of severe hypercortisolism to reduce morbidity and mortality; (b) complete surgical removal of the ACTH-secreting tumor, the only curative procedure; and (c) management of any metastatic disease [71].

### 6.1. Control of Hypercortisolism

In critically ill ECS patients, hypercortisolism is an emergency that needs to be managed without delay [72]. Minimizing the risk of life-threatening conditions such as opportunistic infections, VTE, severe metabolic derangements, and psychosis [73] is crucial.

#### 6.1.1. Medical Therapy

Medical therapy with adrenal steroidogenesis inhibitors is the cornerstone of rapid biochemical control of hypercortisolism [74].

Metyrapone is rapid-acting (hours) and is most frequently used in initial treatment [75,76]. Ketoconazole is also a potent steroidogenesis inhibitor with a slower onset and a risk of hepatotoxicity [76]. The S-enantiomer, levoketoconazole, may have an improved safety profile [74]. Osilodrostat is a newer, more potent 11β-hydroxylase inhibitor [77]. More than one agent is typically required to be used together to achieve eucortisolism in advanced disease [75].

For non-oral treatment in critically ill patients, intravenous etomidate in sub-hypnotic doses can abruptly lower cortisol levels [72,73]. Mifepristone, a glucocorticoid receptor blocker, can rapidly reverse the clinical effects of excess cortisol but does not lower cortisol levels, and therefore, biochemical monitoring of treatment efficacy is challenging [76,78]. Mitotane, an adrenolytic agent, is long-acting and generally reserved for adrenocortical carcinoma but has been used in benign conditions that lead to hypercortisolism as well [76].

#### 6.1.2. Bilateral Adrenalectomy

Surgery is a definitive and immediate therapy for hypercortisolism and is lifesaving in patients with severe, medically intractable disease or when the ectopic source is occult or unresectable [79,80]. Bilateral adrenalectomy is increasingly performed laparoscopically to reduce morbidity [81,82]. It nevertheless results in lifelong adrenal insufficiency requiring lifelong glucocorticoid and mineralocorticoid replacement. Unlike in Cushing’s disease, where loss of cortisol-mediated negative feedback after bilateral adrenalectomy can lead to progressive growth of a pre-existing pituitary corticotroph tumor (Nelson’s syndrome), this specific risk does not apply to ECS, since no pituitary tumor is present; patients nonetheless require long-term surveillance, as the primary ectopic tumor may persist, recur, or only become radiologically apparent after adrenalectomy [83].

#### 6.1.3. Prevention and Management of Life-Threatening Complications

Because severe, rapid-onset ECS constitutes an endocrine emergency, prevention and management of its most lethal complications should proceed in parallel with biochemical control of hypercortisolism. Prophylaxis against *Pneumocystis jirovecii* pneumonia (e.g., with trimethoprim-sulfamethoxazole) should be considered in patients with severe hypercortisolism, particularly while awaiting definitive treatment, given the profound cortisol-induced lymphopenia and immunosuppression [41,42]. Given the markedly increased risk of venous thromboembolism associated with hypercortisolism-induced hypercoagulability, pharmacological thromboprophylaxis should be considered in hospitalized patients without contraindications, with a low threshold for imaging in the presence of suggestive symptoms [41,43]. Close monitoring and aggressive correction of hypokalemia, hyperglycemia, and hypertension further reduce perioperative and overall morbidity [37,38,39,40].

### 6.2. Tumor-Directed Therapy

#### 6.2.1. Curative Resection

The only curative treatment for ECS is resection of the ACTH-secreting tumor [71]. Additionally, if the tumor is localized but not metastatic, surgical resection is the first-line treatment and offers the sole chance of a cure [43]. For well-differentiated bronchial NENs, the cure rate following complete resection has been as high as 83% [84].

#### 6.2.2. Therapies for Metastatic or Unresectable Disease

Various therapeutic modalities can be effective, to an extent, for ECS patients with metastatic or unresectable disease; these are shown in Table 5.

## 7. Prognosis and Morbidity

The prognosis for ECS patients is highly variable and predominantly depends on the histology and stage of the primary tumor [89]. Patients can have a very good outcome when they have benign or low-grade, localized ECS-causing NENs such as bronchial carcinoids that are completely resected, with an overall 5-year survival rate of more than 80–90% [90]. In contrast, the prognosis is poor in patients with high-grade, metastatic malignancies like SCLC, with median survival often less than one year [19,29]. For the remaining NENs, prognosis is intermediate and stage-dependent. A recent meta-analysis reported 5-year survival rates of 81% for pulmonary NENs, 50% for thymic NENs, and 40% for pancreatic NENs [90]. Their prognosis is normally favorable, and their 5-year survival rate is about 66%, reflecting the indolent nature of the underlying, unsuspected NEN [90].

Uncontrolled hypercortisolism predicts an adverse outcome regardless of other considerations [91]. It disproportionately increases mortality risk due to cardiovascular complications, venous thromboembolism (VTE), and life-threatening opportunistic infections, particularly in patients with aggressive underlying malignancies [4,41]. Therefore, early and effective management of excess cortisol is crucial for improving survival, regardless of the nature of the tumor [72]. Even after successful treatment and biochemical control, patients may experience long-term morbidity in the form of persistent hypertension, metabolic derangements, osteoporosis, and impaired quality of life, so long-term follow-up is important [92].

## 8. Future Perspectives

The treatment of ECS has improved over time. Various areas hold promise for the improvement of results in these complex patients [6].

Improved diagnosis and localization procedures are needed. New PET tracers, such as CRH receptor-targeting compounds (e.g., 68Ga-CRH PET-CT, currently investigational and evaluated only in small proof-of-concept cohorts), have the potential to allow more accurate discrimination between pituitary and ectopic origins non-invasively and, in certain patients, reduce or eliminate the requirement for BIPSS in the future [70,93]. Enhanced radiomics and artificial intelligence could also enhance the detection power of standard imaging for microscopic lesions [94].

At the clinical level, novel medical treatments are expanding the armamentarium for treating hypercortisolism [74,95]. The second-generation of steroidogenesis inhibitors, osilodrostat and levoketoconazole, provide more options with potentially divergent side-effect profiles [96,97]. One such promising research area is the development of ACTH receptor antagonists, such as the research drug atumelnant (CRN04894), which act directly by blocking the effect of ACTH at the adrenal gland [98]. This approach could potentially offer a highly specific method to control hypercortisolism regardless of its source, possibly with fewer off-target effects compared to steroidogenesis inhibitors.

Finally, improved insight into the molecular pathogenesis of ectopic hormone secretion by NENs is needed. Identifying the specific genetic and epigenetic factors responsible for abnormal POMC expression and ACTH secretion could uncover novel tumor-specific diagnostic and follow-up markers as well as new targets for rational, molecularly guided therapy [12]. Due to the rarity of ECS, multicenter, international networks and registries, like ERCUSYN, will be important for implementing the prospective studies necessary to confirm new diagnostic approaches and test new therapies [99]. Through these collaborative efforts, we may be able to unravel the complexities of this challenging syndrome and improve the lives of affected patients.

## 9. Conclusions

Cushing’s syndrome caused by ectopic ACTH secretion is among the most challenging disorders of clinical endocrinology, considering its rarity, heterogeneous tumor spectrum, and often fulminant presentation. Prompt identification of severe hypercortisolism as an endocrine emergency is essential, as uncontrolled cortisol excess is a major determinant of early morbidity and mortality. Diagnosis requires stepwise assessment, with the integration of biochemical measurement, inferior petrosal sinus sampling, and rapidly developing imaging methods. The only curative treatment is surgical resection of the responsible tumor; however, when resection is not possible or the tumor cannot be localized, medical management and bilateral adrenalectomy serve as salvage therapies. Prognosis depends on tumor stage and histology. Molecular imaging, newer steroidogenesis inhibitors, and ACTH receptor antagonists hold promise for earlier detection and improved management. Multicenter studies and collaborative registries will be needed to optimize results in this uncommon syndrome.

## Figures and Tables

**Table 1:** Common tumor sources in ectopic ACTH syndrome.

Tumor Type	Approximate Frequency	Key References
**Thoracic Tumors**		
Bronchial Neuroendocrine Neoplasm (NEN)/Carcinoid	20–40%	[6,18,19]
Small-Cell Lung Carcinoma (SCLC)	10–40%	[1,6,19]
Thymic Neuroendocrine Neoplasm (NEN)/Carcinoid	5–10%	[20,21,22]
**Abdominal Tumors**		
Pancreatic Neuroendocrine Neoplasm (PNEN)	10–20%	[6,23,24]
Pheochromocytoma/Paraganglioma	2–6%	[6,25]
**Other**		
Medullary Thyroid Carcinoma (MTC)	2–10%	[1,6]
Occult (Source Undiscovered)	8–20%	[1,6,26]

**Table 2:** Features of severe, rapid-onset ECS.

Clinical Feature	Description/Pathophysiology	Key References
Severe skin hyperpigmentation	Caused by very high circulating concentrations of ACTH and other proopiomelanocortin (POMC)-derived peptides that stimulate melanocytes.	[6]
Severe metabolic disorders	Includes treatment-resistant hypertension and profound hypokalemic metabolic alkalosis. Hypokalemia results from the mineralocorticoid effect of very high cortisol concentrations overwhelming the activity of the renal enzyme 11β-hydroxysteroid dehydrogenase type 2 (11β-HSD2), allowing cortisol to activate the mineralocorticoid receptor.	[6,37,38,39,40]
Severe hyperglycemia or diabetes	Marked hyperglycemia that frequently requires high doses of insulin for glycemic control.	[26]
Psychiatric disorders	Includes severe depression, anxiety, emotional lability, and occasionally acute psychosis.	[36]
Life-threatening complications	Patients are predisposed to opportunistic infections (e.g., *Pneumocystis jirovecii* pneumonia), sepsis, and venous thromboembolism (VTE), which represent major causes of mortality. Due to the severe and rapidly progressive clinical presentation, ECS—particularly aggressive forms—should be considered an endocrine emergency requiring immediate diagnosis and management.	[41,42,43]

**Table 3:** Non-invasive biochemical tests used in the evaluation of ECS.

Test	Principle/Mechanism	Diagnostic Interpretation and Limitations	Key References
High-Dose Dexamethasone Suppression Test (HDDST)	Most pituitary corticotroph adenomas suppress ACTH (and therefore cortisol) production in response to high doses of dexamethasone, whereas most ectopic tumors do not.	The diagnostic utility is limited due to considerable overlap. A significant proportion of ECS cases (up to 40%), particularly those caused by indolent bronchial carcinoids, demonstrate suppression, while about 20% of patients with Cushing disease (CD) fail to suppress. Suppression of cortisol by >50–69% is suggestive of CD, but lack of suppression is not definitive for ECS.	[14,18,49,50]
CRH Stimulation Test	Administration of corticotropin-releasing hormone (CRH) typically elicits a brisk rise in ACTH and cortisol in patients with CD. Most ectopic tumors lack CRH receptors and therefore do not respond.	An exaggerated ACTH/cortisol response strongly suggests CD, while a blunted or absent response suggests an ectopic source. However, some neuroendocrine neoplasms (NENs), particularly bronchial carcinoids, may express CRH receptors and respond to stimulation. The current widespread unavailability of CRH for clinical use has rendered this test largely historical in many regions.	[14,18,49,51]
Desmopressin (DDAVP) Test	Proposed as an alternative to the CRH test. Desmopressin stimulates V2 and V3 vasopressin receptors, which are often overexpressed on corticotroph adenomas.	Although most patients with CD show an ACTH/cortisol response, many ectopic neuroendocrine neoplasms also express these receptors, producing false-positive results and limiting specificity for distinguishing CD from ECS. A meta-analysis reported a specificity of only 64% for distinguishing CD from ectopic ACTH syndrome (EAS) based on the ACTH response.	[52,53,54]

**Table 4:** Nuclear medicine modalities for the assessment of ECS.

Imaging Modality	Principle/Target	Clinical Utility and Limitations	Key References
Somatostatin Receptor (SSTR) Imaging	Most well-differentiated neuroendocrine neoplasms (NENs) express somatostatin receptors (SSTRs) on their cell surface. Gallium-68 (68Ga)-labelled SSTR PET/CT using tracers such as DOTATATE, DOTATOC, or DOTANOC targets these receptors.	68Ga-SSTR PET/CT has largely replaced the less sensitive 111In-octreotide scintigraphy. It provides superior spatial resolution and can detect small lesions missed by conventional imaging. Reported sensitivity for detecting the source of ectopic Cushing syndrome (ECS) ranges from 57% to 82%. It is particularly valuable for identifying occult tumors and for staging metastatic disease.	[62,64,65,67,68]
18F-FDG PET/CT	Detects tumors with high glucose metabolism.	Highly sensitive for aggressive, high-grade malignancies such as small-cell lung carcinoma (SCLC). However, it is often negative in the more common indolent, well-differentiated NENs that cause ECS.	[69]
Other PET Tracers	Additional tracers including 18F-DOPA and novel tracers targeting specific receptors.	18F-DOPA PET has been used with some success, although experience remains limited. A novel, still investigational PET tracer targeting the CRH receptor (68Ga-CRH PET-CT), evaluated so far only in small proof-of-concept series and not yet used in routine clinical practice, has shown promise for non-invasively distinguishing pituitary from ectopic ACTH sources.	[64,70]

**Table 5:** Therapies for ECS patients with metastatic or unresectable disease.

Treatment Modality	Mechanism/Principle	Clinical Use and Limitations	Key References
Somatostatin Analogues (SSAs)	Long-acting somatostatin analogues such as octreotide and lanreotide act on somatostatin receptors (SSTRs) expressed by neuroendocrine tumors.	Used in patients with SSTR-positive tumors to control symptoms related to secretion of other peptides and to exert some anti-proliferative effects. However, when used alone, they are not effective in controlling ACTH-mediated hypercortisolism.	[73,85]
Chemotherapy	Cytotoxic chemotherapy targeting rapidly proliferating tumor cells.	Preferred treatment for high-grade tumors such as small-cell lung carcinoma (SCLC), typically using regimens such as etoposide and cisplatin. Also used in metastatic, poorly differentiated neuroendocrine neoplasms (NENs).	[29]
Peptide Receptor Radionuclide Therapy (PRRT)	Targeted radionuclide therapy using radiolabeled somatostatin analogues (e.g., 177Lutetium-DOTATATE) that bind to SSTRs on tumor cells and deliver cytotoxic radiation.	Used in metastatic, well-differentiated neuroendocrine neoplasms with high somatostatin receptor uptake on functional imaging to control tumor growth.	[86]
Targeted Molecular Therapies	Molecularly targeted kinase inhibitors directed at specific oncogenic drivers.	In selected tumor types such as medullary thyroid carcinoma (MTC) with RET mutations, targeted inhibitors (e.g., selpercatinib) are effective and may also reduce ectopic ACTH secretion.	[87]
Radiotherapy	External beam radiotherapy delivering localized ionizing radiation.	Used for palliative treatment of symptomatic metastases (e.g., bone metastases) or for localized unresectable tumors.	[88]

## Data Availability

No new data were created or analyzed in this study. Data sharing is not applicable to this article.

## Abbreviations

Abbreviation	Full term
ACTH	Adrenocorticotropic hormone
BIPSS	Bilateral inferior petrosal sinus sampling
CD	Cushing’s disease
CRH	Corticotropin-releasing hormone
CS	Cushing’s syndrome
CT	Computed tomography
DDAVP	Desmopressin
ECS	Ectopic Cushing’s syndrome
EAS	Ectopic ACTH syndrome
ERCUSYN	European Registry on Cushing’s Syndrome
FDG	Fluorodeoxyglucose
GEP-NETs	Gastroenteropancreatic neuroendocrine tumors
HDDST	High-dose dexamethasone suppression test
HPA axis	Hypothalamic–pituitary–adrenal axis
IPS:P	Inferior petrosal sinus-to-peripheral ACTH ratio
LNSC	Late-night salivary cortisol
MEN-1	Multiple Endocrine Neoplasia Type 1
MEN-2	Multiple Endocrine Neoplasia Type 2
MRI	Magnetic resonance imaging
MTC	Medullary thyroid carcinoma
NEN	Neuroendocrine neoplasm
NET	Neuroendocrine tumor
ODST	Overnight dexamethasone suppression test
PET	Positron emission tomography
PET/CT	Positron emission tomography–computed tomography
PNEN	Pancreatic neuroendocrine neoplasm
POMC	Pro-opiomelanocortin
PRRT	Peptide receptor radionuclide therapy
RET	Rearranged during transfection (proto-oncogene)
SCLC	Small-cell lung carcinoma
SSA	Somatostatin analogue
SSTR	Somatostatin receptor
UFC	Urinary free cortisol
VTE	Venous thromboembolism
11β-HSD2	11β-Hydroxysteroid Dehydrogenase Type 2
68Ga	Gallium-68
111In	Indium-111
18F-FDG	Fluorine-18 Fluorodeoxyglucose
18F-DOPA	Fluorine-18 Dihydroxyphenylalanine
